# The injury epidemiology of cyclists based on a road trauma registry

**DOI:** 10.1186/1471-2458-11-653

**Published:** 2011-08-17

**Authors:** Emmanuelle Amoros, Mireille Chiron, Bertrand Thélot, Bernard Laumon

**Affiliations:** 1 Epidemiological Research and Surveillance Unit in Transport, Occupation and Environment (UMRESTTE), Université de Lyon, 43 bvd du 11 Novembre 1918 F-69622 Lyon, France; 2 Epidemiological Research and Surveillance Unit in Transport, Occupation and Environment (UMRESTTE), French Institute of Science and Technology for Transport, Development and Networks (IFSTTAR), 25 avenue François Mitterrand, BRON, F-69675, France; 3 Epidemiological Research and Surveillance Unit in Transport, Occupation and Environment (UMRESTTE), Université Lyon 1, Lyon, F-69373, France; 4 Injury Unit, Department of Chronic Diseases and Trauma, French Institute for Public Health Surveillance (InVS), 12 rue du Val d'Osne, Saint-Maurice, F-94415, France

## Abstract

**Background:**

Bicycle use has increased in some of France's major cities, mainly as a means of transport. Bicycle crashes need to be studied, preferably by type of cycling. Here we conduct a descriptive analysis.

**Method:**

A road trauma registry has been in use in France since 1996, in a large county around Lyon (the Rhône, population 1.6 million). It covers outpatients, inpatients and fatalities. All injuries are coded using the Abbreviated Injury Scale (AIS). Proxies were used to identify three types of cycling: learning = children (0-10 years old); sports cycling = teenagers and adults injured outside towns; cycling as means of transport = teenagers and adults injured in towns. The study is based on 13,684 cyclist casualties (1996-2008).

**Results:**

The percentage of cyclists injured in a collision with a motor vehicle was 8% among children, 17% among teenagers and adults injured outside towns, and 31% among those injured in towns. The percentage of serious casualties (MAIS 3+) was 4.5% among children, 10.9% among adults injured outside towns and 7.2% among those injured in towns. Collisions with motor-vehicles lead to more internal injuries than bicycle-only crashes.

**Conclusion:**

The description indicates that cyclist type is associated with different crash and injury patterns. In particular, cyclists injured in towns (where cycling is increasing) are generally less severely injured than those injured outside towns for both types of crash (bicycle-only crashes and collisions with a motor vehicle). This is probably due to lower speeds in towns, for both cyclists and motor vehicles.

## Background

In some of France's major cities (Paris, Lyon, Lille,...) there has been an increase in cycling, mostly as a means of transport [1]. This is partly associated with local policies, such as the introduction of large self-service bicycle sharing schemes. More generally, cycling as a means of transport is encouraged in the framework of sustainable development. We therefore need to know more about cyclist road risk, according to the type of cycling: as a leisure or sporting activity or as a means of transport. These three types are fairly common now in France and they seem to have different risk patterns [2]. As a first step, we conducted here a descriptive study of cyclists' crash characteristics and cyclists' injuries, by type of cycling.

Police crash data are not appropriate for studying injured cyclists, because of specific strong under-reporting and selection bias on this road user type [3]. In France, the probability of injured cyclists being recorded in the police data has been estimated at 34% for collisions with motor vehicles and at about 2% for bicycle-only crashes [4]. In this study we have used data from a road trauma registry. This is much more complete than police data, with an estimated coverage rate for injured cyclists of about 80% [5]. This study involves a total of 13,684 injured cyclists (who could be classified into a given type of cyclists, among 16,849 recorded ones). The registry contains detailed medical information on the injuries, which have been coded using the Abbreviated Injury Scale (AIS) [6].

The objective of this study is to improve our knowledge of cyclists' crashes and injuries, according to the type of cyclist. We have described the circumstances of the crash (month, day, crash opponent...), the characteristics of the cyclists (age and gender) and the nature of the cyclists' injuries (severity, types and affected body regions).

## Methods

### Data

The Rhône county has a population of 1.6 million inhabitants and includes a large city (Lyon) with its suburbs and a rural area. A road trauma registry has been in operation in this county since 1996 [7]. It covers all the casualties from road crashes occurring in the Rhône county who sought medical care in health facilities and covers the whole range of injury severity: outpatients, inpatients and fatalities. All the healthcare facilities (in public and private hospitals) in the county and its surrounding area which receive crash victims contribute to the registry, i.e. about 260 health departments ranging from pre-hospital emergency care, emergency departments, intensive care units, surgery units to rehabilitation departments. The forensic medicine institutes provide data on those killed at the scene. Injury assessment is based on all the diagnoses made in the different health services the casualty passed through. Each injury is coded with the Abbreviated Injury Scale, 1990 revision [6]. Codes consist of the body region (R), the type of anatomical structure (T), the specific anatomic structure (S) and the level of injury (N). An immediate severity score, known as the AIS score, is ascribed to each injury code. This has 6 levels: 1 (minor), 2 (moderate), 3 (serious), 4 (severe), 5 (critical) and 6 (beyond treatment). In this study, in order to measure the whole body injury severity of a casualty, we have used the MAIS (Maximum AIS). This is the severity score of the subject's most severe injury. An injury impairment score, between 0 and 6, is also assigned to each injury code. This is the Injury Impairment Scale, which was proposed by Hirsh and Eppinger [8] in work for the Association for the Advancement of Automotive Medicine (AAAM). The IIS values were assigned on the basis of consensus between 35 experts. They take account of mobility, cognitive capacities, aesthetic, sensory or sexual impairment and/or pain. In order to measure the whole-body impairment of each casualty, we have used the Maximum IIS (MIIS), which is the highest IIS of his/her injuries. Over the 1996-2008 period, the Rhône road trauma registry recorded 16,849 injured cyclists, 63 of whom were killed.

The study is based on data from the Rhône road trauma registry only. This registry has been certified by the Comité National des Registres (CNR); this commission evaluates the scientific and ethical aspects of registries in France. This registry has also been approved by the Commission Nationale Informatique et Libertés (CNIL). This commission evaluates any recording of data and its use, in terms of ethics. The approval by the CNIL implies in particular the right for conducting statistical analyses (on ethically collected data).

### Method

We began by calculating incidences of injured cyclists, according to age and gender, and of hospitalised cyclists, per 100,000 inhabitants, based on the population of the Rhône county.

We wished to identify different groups of cyclists according to their type of bicycle use. The first such group we identified was children (0-10 year old), who are learner cyclists. For other cyclists, i.e. teenagers and adults, we wished to distinguish between sports or leisure cycling and cycling as a means of transport. Since this information does not figure in the trauma registry, we used the location of the crash, making a distinction between 'outside towns' and 'in towns', using crash location as a proxy for cycling location. Cycling area was in turn considered as a proxy for the type of cycling: most sports or leisure cycling takes place 'outside towns', and most cycling as a means of transport takes place 'in towns'. We distinguished between the two types of location on the basis of the ZAUER classification, created by INSEE - the French National Institute of Statistics and Economic Studies - population density and population size. We defined 'in towns' as being a "rural employment cluster" (more than 5000 jobs) or an "urban cluster" that we further restricted to those with a population density of over 500 persons per km^2 ^or a population of over 5,000. All other locations were considered to be 'outside towns'. Our division of casualties into those injured "in towns" and those injured "outside towns" also means our findings do not depend on the urbanization rate of the Rhône department, and can hence be generalised.

We have provided a descriptive analysis that compares the different types of cyclist. This analysis is based on a subsample of 13,684 cyclists, as 18.8% of the 16,849 injured cyclists could not be classified because of an unknown crash location. This subsample contained 3,671 injured children (0-10 years old), 2,032 'teenagers and adults injured outside towns', and 7,981 'teenagers and adults injured in towns'.

We have described the crash characteristics for each type of cyclist (type of road, day and month of crash, time of day (day/night), type of crash opponent if any and type of trip), as well as the characteristics of the cyclists (gender, age), and the characteristics of the injuries sustained.

We have displayed the injury patterns of all AIS 2+ injuries, ignoring minor injuries (AIS 1; mostly abrasions and contusions). These AIS 2+ injury patterns were described according to the type of cyclist and separately for collisions with a motor vehicle and bicycle-only crashes (no crash opponent). A distinction was made between these two types of accident as they lead to quite different injury patterns. The injury pattern was obtained by displaying the distribution of injuries according to both injured body region and injury type, in a matrix that closely resembles the Barell matrix [9]. The following body regions were used: head; face; neck; unspecified region of the head, face or neck; torso; vertebral column; upper extremities; lower extremities; system-wide or unspecified. The types of injury were divided into the following categories: fractures; dislocations, sprains and strains; internal organ injuries (including fractures combined with internal organ or blood vessels injuries); open wounds; contusions and abrasions (but there is none among AIS 2+injuries); unspecified; others (burns, nerve injuries, blood vessel injuries, crushing and amputations). 'Unspecified' and 'others' were not grouped together as 'unspecified head injury' is fairly informative as it means 'unconsciousness without further injury description'; the 'others' category contains injuries that are extremely rare among the cyclists. The statistical unit in these tables is the injury, not the casualty. This implies that we take into account the different injuries a casualty may have.

A detailed description, according to type of cyclist were provided for potentially fatal injuries (AIS 4, 5 or 6)

Note: In this paper, the term 'injured cyclists' includes the 63 that were killed.

## Results and discussion

### Incidence

The mean incidence of injured cyclists over the period 1996-2008 was 80/100,000. During this period the incidence fell from 99/100,000 in 1996 to 70/100,000 in 2008. This is not due to a decrease in cycling; on the contrary, cycling use was multiplied by 3 between 1995 and 2006 in the area of Lyon and around -which does not exactly correspond to the Rhône area but nearly- [10]. On the national level, cycling use has been stable [10]. The decrease in incidence of road crashes has in fact been observed for all road user types in France (except motorized two-wheeler users) following the large-scale deployment of automatic speed control cameras in 2003 [11]. The mean incidence of hospitalized cyclists in the period was 14/100,000.

**Figure 1:**
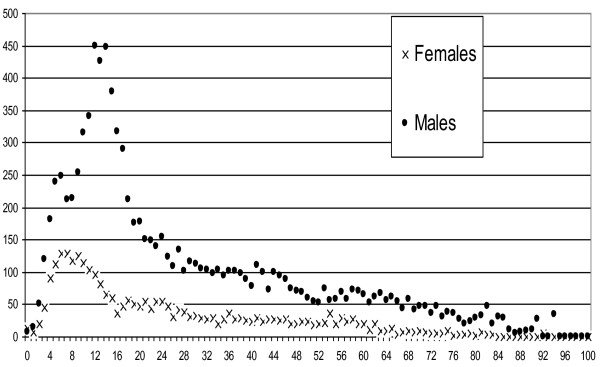
**incidence (per 100,000 inhabitants) of cycling casualties (injured or killed), according to age and gender (Rhône Road Trauma Registry, 1996-2006, n = 14437)**.

The incidence was much higher for men than for women, the mean sex-ratio being 3.6 (Figure 1). The incidence peaked in children, at 12-14 years of age for boys and 7-9 years of age for girls. These are very consistent with the pattern of exposure, in other words with the pattern of cycling according to age and gender as revealed by the French national transport survey [12]: women account for between 15% and 25% of sports cycling trips and between 30 and 44% of non-sports cycling trips. The higher incidence in males may also be due to stronger risk-taking behaviour in comparison with females [13,14]. The higher incidence of bicycle injuries among men than women has also been observed in other countries [15-17].

The overall incidence was of the same order of magnitude as in Australia, where the incidence of injured cyclists who visited emergency departments was 122/100,000 [18]. But it was far below that observed in Sweden, where the available incidence, 398/100,000, is admittedly from the region where it is probably the highest [19,20]. The incidence of bicycle injuries is of course associated with the amount of bicycle use and this is much higher in Scandinavian countries than in France.

### Descriptive analysis of cyclist casualties, according to cyclist type

#### Crash characteristics

The distribution of injured cyclists according to the month of their crash (Table 1) is non-uniform with most crashes occurring between April and September, reflecting the pattern of exposure: more cycling in this period than in winter. The interesting point is a slightly greater range between winter and spring-summer for children (a range between 1.3% and 14.8%) and for 'teenagers and adults injured outside towns' (range between 2.5% and 12.5%) than for 'teenagers and adults injured in towns' (range between 3.8% and 11.7%)

**Table 1:** Crash characteristics according to cyclist type, Rhône Road Trauma Registry, 1996-2008, n = 13,684

		Teens & adults, injured in towns	Teens & adults, injured outside towns	Children (0-10 years old)
		N = 7,981	N = 2,032	N = 3,671
		**Col. %**	**Col. %**	**Col. %**

Month of crash	January	4.7%	2.5%	2.2%
p < 0.0001	February	4.3%	4.1%	2.8%
	March	6.4%	6.1%	7.3%
	April	8.7%	10.2%	9.9%
	May	11.4%	11.9%	14.8%
	June	11.4%	12.5%	11.5%
	July	11.7%	12.3%	11.5%
	August	10.1%	11.4%	14.7%
	September	11.8%	12.1%	13.9%
	October	9.4%	9.8%	7.2%
	November	6.3%	4.1%	2.7%
	December	3.8%	3.1%	1.3%

Day of crash	Monday	12.6%	8.8%	11.4%
p < 0.0001	Tuesday	14.0%	10.0%	10.5%
	Wednesday	15.7%	11.9%	15.0%
	Thursday	13.6%	11.7%	10.5%
	Friday	14.6%	8.8%	11.1%
	Saturday	14.1%	21.5%	18.3%
	Sunday	15.4%	27.4%	23.3%

Time of day	Day time	62.0%	82.7%	63.2%
p < 0.0001	Night time	12.1%	3.9%	2.4%
	Unknown	25.9%	13.3%	34.4%

Road type	'National roads'	0.8%	3.3%	0.1%
p < 0.0001	'County roads'	1.2%	16.1%	1.1%
	'Local roads'	73.3%	39.5%	43.3%
	Road, unknown type	2.9%	5.1%	2.5%
	Car park	0.9%	0.7%	2.0%
	Private road	1.0%	1.2%	6.0%
	Off-road	7.3%	13.7%	9.4%
	Unknown	12.4%	20.2%	35.6%

Type of trip	Private	33.0%	42.3%	0.0%
p < 0.0001	Work-related*	14.3%	2.6%	0.0%
	Not relevant**	29.3%	34.1%	100.0%
	Unknown	23.4%	21.1%	0.0%

Antagonist type p < 0.0001	None or non-motorized	67.1%	80.6%	89.6%
	Motor vehicle	30.9%	16.5%	7.6%
	Unknown	2.0%	3.0%	2.8%

Antagonist type	None	54.8%	68.0%	78.9%
(detail)	Stationary	7.9%	5.9%	6.5%
p < 0.0001	Animal	0.3%	0.4%	0.0%
	Pedestrian	1.1%	0.6%	0.7%
	Cyclist	3.1%	5.7%	3.5%
	Motorised two wheeler	1.2%	1.3%	0.4%
	Car	27.7%	13.7%	6.8%
	Van, Truck	2.0%	1.5%	0.4%
	Unknown	2.0%	3.0%	2.8%

* work-related = commuting or part of occupational activity

** not relevant = outside the range 14 to 65 years old

An interesting pattern is observed for the day of the crash. There is a non-uniform distribution with a peak on Wednesdays (when primary schools are closed) and at week-ends for injured children (23.3% of crashes occurred on Sunday), a peak at week-ends for 'teenagers and adults injured outside towns' (27.4% on Sunday), but a uniform distribution for 'teenagers and adults injured in towns' (15.4% on Sunday). This is consistent with the cycling patterns [12]: sports cyclists account for 34% of bicycle trips on Sundays, 22% on Saturdays and 13% on week days.

With regard to the time of day, the distribution of cyclist injuries between the day and night shows that it was quite rare for children to be injured at night and the same applies to 'teenagers and adults injured outside towns', but it was not so rare for cyclists injured in towns. This is also probably due to more cycling at night for this group, since cycling in town corresponds mostly to using the bicycle as a means of transport.

The distribution of injured cyclists according to type of road reveals a very different distribution across cyclist types. Obviously, the crashes that occurred in towns were mostly on streets (73.3%) while a nontrivial proportion (19.4%) of the 'teenagers and adults injured outside towns' were injured on a major road ('national' and 'county' roads).

The proportion of crashes that occurred during a work-related trip (vs a private trip) also varied with cyclist type. It was higher among 'teenagers and adults injured in towns' (14.3%) than among those 'injured outside towns' (2.6%) (Note: although a non-trivial proportion of trips were of unknown type, the percentages were similar for both types of cyclist, respectively 23.4% and 21.1%, so that the comparison is possible).

The proportion of bicycle-only crashes varied with cyclist type, from 62.7% for 'teenagers and adults injured in towns', to 73.9% for those 'injured outside towns' and 85.4% for injured children. In slightly different terms, the proportion of crashes which involved a motor vehicle was as low as 8% among injured children, and rose to one third in 'teenagers and adults injured in towns'. Again, this is probably related to exposure to different amounts of motorised traffic. The most frequent opponent was a car (for all cyclist types).

The construction of 'cyclist type', using age and crash location as proxies, appears to be relevant: the crash circumstances of the three cyclist groups differed and they differed in ways which corresponded to the available information on cycling patterns [12].

#### Cyclists' characteristics

The proportion of females among the injured cyclists was highest among children (31.6%), and lowest among 'teenagers and adults injured outside towns' (15.3%). The 'teenagers and adults injured outside towns' were somewhat older than those injured 'in towns'.

With regard to injury severity (Table 2), 'teenagers and adults injured outside towns' were the group that was most often seriously injured (10.9% with MAIS 3+), followed by 'teenagers and adults injured in towns' (7.2% with MAIS 3+), and lastly by children (4.5% with MAIS 3+).

**Table 2:** cyclists and injuries characteristics according to cyclist type, Rhône road trauma registry, 1996-2008, n = 13,684

		Teens & adults injured in towns	Teens & adults injured outside towns	Children (0-10 years old)
		N = 7,981	N = 2,032	N = 3,671
		**Col. %**	**Col. %**	**Col. %**

Age	0-4 years old	0.0%	0.0%	19.9%
p < 0.001	5-9 years old	0.0%	0.0%	64.3%
	10-14 years old	20.4%	24.7%	15.8%
	15-19 years old	16.6%	14.0%	0.0%
	20-24 years old	12.4%	5.7%	0.0%
	25-34 years old	17.6%	13.0%	0.0%
	35-44 years old	12.6%	15.9%	0.0%
	45-54 years old	9.6%	12.4%	0.0%
	55-64 years old	5.8%	9.0%	0.0%
	65-74 years old	3.5%	4.1%	0.0%
	75 + years old	1.4%	1.0%	0.0%
	unknown	0.1%	0.2%	0.0%

Gender	female	23.3%	15.3%	31.6%
p < 0.001	male	76.6%	84.7%	68.4%

Number of injuries	1	45.2%	41.2%	63.4%
p < 0.001	2	31.5%	31.3%	26.8%
	3	16.2%	17.6%	7.5%
	4	5.0%	5.9%	1.9%
	5 or more	2.1%	4.0%	0.5%

Injury severity	MAIS 1 (slight)	64.7%	54.4%	73.6%
p < 0.0001	MAIS 2 (moderate)	28.1%	34.7%	21.9%
	MAIS 3 (serious)	5.6%	8.2%	4.2%
	MAIS 4 (severe)	0.9%	1.6%	0.3%
	MAIS 5 (critical)	0.2%	0.3%	.
	MAIS 6 or killed	0.6%	0.7%	0.1%

Injury impairment	MIIS 0 (none)	79.1%	74.5%	89.0%
	MIIS 1 (slight)	19.0%	22.5%	10.5%
	MIIS 2 (moderate)	0.9%	1.4%	0.2%
	MIIS 3 (serious)	0.3%	0.6%	0.1%
	MIIS 4 (severe)	0.4%	0.6%	0.1%
	MIIS 5 (critical)	0.1%	0.1%	0.0%
	MIIS 6	0.1%	0.2%	0.0%

Medical care	Emergency Dpt. only	81.8%	73.8%	82.7%
p < 0.0001	Hospitalized	17.8%	25.7%	17.3%
	Deceased without hospitalization	0.4%	0.4%	0.0%

The pattern was similar with regard to impairment: the proportion of injured cyclists with predicted impairment one year after the crash was 25.5% for 'teenagers and adults injured outside towns', 20.9% for those injured in towns, and 11.0% for children.

The highest hospitalization rate was for 'teenagers and adults injured outside towns' at 26% versus a rate of 18% for both children and 'teenagers and adults injured in towns'.

#### Injury patterns

The types of injury and injured body regions for 'teenagers and adults injured in towns' are provided in Tables 3 and 4. For those injured in bicycle-only crashes (Table 3), fractures of the upper extremities accounted for a high percentage (50% of all their AIS 2+ injuries); unspecified head injuries (unconsciousness without any further specified injury) accounted for 13.5%, and internal organ injuries for 5.5% (3.6% to the head and 1.7% to the torso). The injury pattern for 'teenagers and adults injured in towns' in a collision with a motor vehicle was different (Table 4): among their AIS 2+ injuries, there was a much lower percentage of fractures of the upper extremities (25.6% vs 50%), a higher percentage of fractures of the lower extremities, i.e. 16.7% (vs 10.1%), a much higher percentage of internal organ injuries, i.e. 17.0% vs 5.5% (8.4% to the head and 7.6% to the torso), and a slightly higher percentage of unspecified head injuries, i.e. 16.0% (vs 13.5%).

**Table 3:** AIS 2+ injuries of 'teenagers and adults injured in towns', in bicycle-only crashes, (n = 2490 injuries, mean number of injuries per cyclist = 1.25), Rhône Road Trauma Registry, 1996-2008

	Fractures	Dislocations, Sprains and Strains	Internal organ injuries	Open wounds	Unspecified	Other	All types
Head	1.9%	0.0%	3.6%	0.4%	13.5%	0.0%	19.4%
Face	2.4%	0.0%	0.0%	1.1%	0.0%	0.0%	3.5%
Neck	0.0%	0.0%	0.0%	0.1%	0.0%	0.2%	0.3%
*Head face, neck unspec*.	*0.0 *%	*0.0 *%	*0.0 *%	*0.0 *%	*0.0 *%	*0.0 *%	*0.0 *%
Torso	1.8%	0.0%	1.7%	0.1%	0.0%	0.0%	3.6%
Vertebral column	2.0%	0.1%	0.2%	0.0%	0.0%	0.0%	2.3%
Upper extremities	50.7%	3.5%	0.0%	0.9%	0.4%	0.0%	55.6%
Lower extremities	10.1%	3.6%	0.0%	1.5%	0.0%	0.0%	15.2%
*System-wide or unspec*.	*0.0 *%	*0.0 *%	*0.0 *%	*0.0 *%	*0.0 *%	*0.0 *%	*0.0 *%

All regions	69.0%	7.2%	5.5%	4.0%	14.0%	0.3%	100.0%

Each cell shows the proportion of AIS 2+ injuries of the corresponding category among all AIS2+ injuries of the subgroup (teens & adults injured in towns in bicycle-only crashes); the statistical unit is the injury, not the casualty

**Table 4:** AIS 2+ injuries of 'teenagers and adults injured in towns', in collisions with a motor vehicle (n = 1282 injuries, mean number of injuries per cyclist = 1.64), Rhône Road Trauma Registry, 1996-2008

	Fractures	Dislocations, Sprains and Strains	Internal organ injuries	Open wounds	Unspecified	Other	All types
Head	2.7%	0.0%	8.4%	0.8%	16.0%	0.2%	28.0%
Face	3.1%	0.1%	0.0%	0.6%	0.0%	0.1%	3.9%
Neck	0.1%	0.0%	0.0%	0.0%	0.0%	0.3%	0.4%
*Head face, neck unspec*.	*0.0 *%	*0.0 *%	*0.5 *%	*0.0 *%	*0.0 *%	*0.0 *%	*0.5 *%
Torso	3.1%	0.0%	7.6%	0.2%	0.0%	0.4%	11.2%
Vertebral column	5.3%	0.2%	0.4%	0.0%	0.1%	0.0%	6.0%
Upper extremities	25.6%	1.6%	0.0%	0.8%	0.2%	0.1%	28.2%
Lower extremities	16.7%	3.1%	0.1%	1.4%	0.0%	0.4%	21.7%
*System-wide or unspec*.	*0.0 *%	*0.0 *%	*0.0 *%	*0.0 *%	*0.0 *%	*0.0 *%	*0.0 *%

All regions	56.6%	5.1%	17.0%	3.7%	16.2%	1.4%	100.0%

Each cell shows the proportion of AIS 2+ injuries of the corresponding category among all AIS2+ injuries of the subgroup (teens & adults injured in towns in collisions with a motor-vehicle); the statistical unit is the injury, not the casualty

The 'teenagers and adults injured outside towns' who were involved in bicycle-only crashes (Table 5) differed from those injured in towns in the same type of crash, with a higher share of injuries to the internal organs (9.3% vs 5.5%). 'Teenagers and adults injured outside towns' who were involved in collisions with a motor vehicle (Table 6) had an injury pattern that was similar to those injured in towns, but with a higher share of internal injuries to the torso (11.2% vs 7.6%) and a higher share of vertebral fractures (11.2% vs 5.3%).

**Table 5:** AIS 2+ injuries of 'teenagers and adults injured outside towns' in bicycle-only crashes (n = 992 injuries, mean number of injuries per cyclist = 1.34), Rhône Road Trauma Registry, 1996-2008

	Fractures	Dislocations, Sprains and Strains	Internal organ injuries	Open wounds	Unspecified	Other	All types
Head	1.6%	0.0%	6.0%	0.3%	15.3%	0.1%	23.4%
Face	4.1%	0.0%	0.0%	1.1%	0.0%	0.0%	5.2%
Neck	0.0%	0.0%	0.0%	0.0%	0.0%	0.0%	0.0%
*Head face, neck unspec*.	*0.0 *%	*0.0 *%	*0.0 *%	*0.0 *%	*0.0 *%	*0.0 *%	*0.0 *%
Torso	2.1%	0.0%	3.0%	0.1%	0.0%	0.0%	5.2%
Vertebral column	3.1%	0.0%	0.2%	0.0%	0.0%	0.0%	3.3%
Upper extremities	43.1%	4.7%	0.0%	1.0%	0.6%	0.1%	49.6%
Lower extremities	10.5%	1.0%	0.0%	1.6%	0.0%	0.1%	13.2%
*System-wide or unspec*.	*0.0 *%	*0.0 *%	*0.0 *%	*0.0 *%	*0.0 *%	*0.0 *%	*0.0 *%

All regions	64.6%	5.7%	9.3%	4.1%	15.9%	0.3%	100.0%

Each cell shows the proportion of AIS 2+ injuries of the corresponding category among all AIS2+ injuries of the subgroup (teens & adults injured outside towns in bicycle-only crashes); the statistical unit is the injury, not the casualty

**Table 6:** AIS2+ injuries of 'teenagers and adults injured outside towns' in collisions with a motor vehicle (n = 366 injuries, mean number of injuries per cyclist = 2.30), Rhône Road Trauma Registry, 1996-2008

	Fractures	Dislocations, Sprains and Strains	Internal organ injuries	Open wounds	Unspecified	Other	All types
Head	2.5%	0.0%	9.3%	0.8%	10.7%	0.0%	23.2%
Face	1.9%	0.0%	0.0%	0.8%	0.0%	0.0%	2.7%
Neck	0.0%	0.0%	0.0%	0.0%	0.0%	0.3%	0.3%
*Head face, neck unspec*.	*0.0 *%	*0.0 *%	*0.0 *%	*0.0 *%	*0.0 *%	*0.0 *%	*0.0 *%
Torso	1.9%	0.0%	11.2%	0.0%	0.0%	0.0%	13.1%
Vertebral column	11.2%	0.0%	0.3%	0.0%	0.0%	0.0%	11.5%
Upper extremities	23.8%	1.6%	0.0%	1.1%	0.0%	0.3%	26.8%
Lower extremities	18.6%	1.4%	0.0%	1.6%	0.0%	0.5%	22.1%
*System-wide or unspec*.	*0.0 *%	*0.0 *%	*0.0 *%	*0.0 *%	*0.0 *%	*0.3 *%	*0.3 *%

All regions	59.8%	3.0%	20.8%	4.4%	10.7%	1.4%	100.0%

Each cell shows the proportion of AIS 2+ injuries of the corresponding category among all AIS2+ injuries of the subgroup (teens & adults injured outside towns in collisions with a motor-vehicle); the statistical unit is the injury, not the casualty

Children injured in bicycle-only crashes (Table 7) were slightly less severely injured than 'teenagers and adults injured in towns' in crashes of this type, with a smaller proportion of injuries to the internal organs (3.1% vs 5.5%) and a very high proportion of fractures of the upper extremities (65.4% of all their AIS 2+ injuries). Children injured in collisions with a motor vehicle (Table 8) showed a different injury pattern from 'teenagers and adults injured in towns' in crashes of this type, with a slightly higher percentage of injuries to the internal organs (22.1% vs 17.0%) especially to the head (15.3% vs 8.4%), a slightly higher percentage of head fractures (4.6% vs 2.7%) but a lower percentage of vertebral fractures (1.5% vs 5.3%).

**Table 7:** AIS2+ injuries of children injured in bicycle-only crashes (n = 999 injuries, mean number of injuries per cyclist = 1.17), Rhône Road Trauma Registry, 1996-2008

	Fractures	Dislocations, Sprains and Strains	Internal organ injuries	Open wounds	Unspecified	Other	All types
Head	0.9%	0.0%	1.5%	0.4%	14.2%	0.1%	17.1%
Face	1.2%	0.1%	0.0%	2.7%	0.0%	0.0%	4.0%
Neck	0.0%	0.0%	0.0%	0.0%	0.0%	0.1%	0.1%
*Head face, neck unspec*.	*0.0 *%	*0.0 *%	*0.0 *%	*0.0 *%	*0.0 *%	*0.0 *%	*0.0 *%
Torso	0.2%	0.0%	1.6%	0.0%	0.0%	0.0%	1.8%
Vertebral column	0.0%	0.0%	0.0%	0.0%	0.0%	0.0%	0.0%
Upper extremities	65.4%	0.1%	0.0%	0.5%	0.0%	0.1%	66.1%
Lower extremities	7.9%	1.1%	0.0%	1.9%	0.0%	0.0%	10.9%
*System-wide or unspec*.	*0.0 *%	*0.0 *%	*0.0 *%	*0.0 *%	*0.0 *%	*0.0 *%	*0.0 *%

All regions	75.6%	1.3%	3.1%	5.5%	14.2%	0.3%	100.0%

Each cell shows the proportion of AIS 2+ injuries of the corresponding category among all AIS2+ injuries of the subgroup (children injured in bicycle-only crashes); the statistical unit is the injury, not the casualty

**Table 8:** AIS2+ injuries of children injured in collisions with a motor vehicle (n = 131 injuries, mean number of injuries per cyclist = 1.51), Rhône Road Trauma Registry, 1996-2008

	Fractures	Dislocations, Sprains and Strains	Internal organ injuries	Open wounds	Unspecified	Other	All types
Head	4.6%	0.0%	15.3%	0.0%	24.4%	0.0%	44.3%
Face	1.5%	0.0%	0.0%	0.8%	0.0%	0.0%	2.3%
Neck	0.0%	0.0%	0.0%	0.0%	0.0%	0.0%	0.0%
*Head face, neck unspec*.	*0.0 *%	*0.0 *%	*0.8 *%	*0.0 *%	*0.0 *%	*0.0 *%	*0.8 *%
Torso	0.8%	0.0%	6.1%	0.0%	0.0%	0.0%	6.9%
Vertebral column	1.5%	0.0%	0.0%	0.0%	0.0%	0.0%	1.5%
Upper extremities	21.4%	0.0%	0.0%	0.8%	0.0%	0.0%	22.1%
Lower extremities	19.1%	1.5%	0.0%	1.5%	0.0%	0.0%	22.1%
*System-wide or unspec*.	*0.0 *%	*0.0 *%	*0.0 *%	*0.0 *%	*0.0 *%	*0.0 *%	*0.0 *%

All regions	48.9%	1.5%	22.1%	3.1%	24.4%	0.0%	100.0%

Each cell shows the proportion of AIS 2+ injuries of the corresponding category among all AIS2+ injuries of the subgroup (children injured collisions with a motor-vehicle); the statistical unit is the injury, not the casualty

'Teenagers and adults injured outside towns' sustained more injuries on average than 'teenagers and adults injured in towns', who in turn sustained more injuries on average than children.

It is interesting to note that 'teenagers and adults injured in towns' were on the whole less severely injured than 'teenagers and adults injured outside towns', even though the former were more often involved in a collision with a motor vehicle than the latter, and collisions with a motor vehicle are more severe than bicycle-only crashes. This can be explained by lower speeds in towns, both for the cyclists and the motor vehicles.

The potentially fatal (AIS 4+) injuries are presented in Table 9, for each type of cyclist. For this level of severity, the injury patterns for 'teenagers and adults injured in towns' and 'teenagers and adults injured outside towns' appear to be similar. About two-thirds of the severe injuries were to the head, about 20% to the thorax, and the remainder to the abdomen and the spine. The most common injuries to the head were haematomas of the cerebrum. The children seem to have mainly sustained head injuries, but this conclusion is based on a very low frequency (14 injuries).

**Table 9:** Description of possibly fatal (AIS4+) injuries, by type of cyclist, Rhône road trauma registry, 1996-2008

Teenagers and adults injured in towns		Teenagers and adults injured outside towns		Injured children		Total		
**n = 182 injuries**		**n = 77 injuries**		**n = 14 injuries**		**n = 273 injuries**		
**n**	**Col. %**	**n**	**Col. %**	**n**	**Col. %**	**n**	**Col. %**	

								**head**
7	3.8%	0	0.0%	1	7.1%	8	2.9%	whole area, major injury or massive destruction
1	0.5%	0	0.0%	0	0.0%	1	0.4%	vertebral artery, laceration
3	1.6%	4	5.2%	0	0.0%	7	2.6%	brain stem, contusion, hemorrhage or laceration
1	0.5%	0	0.0%	0	0.0%	1	0.4%	cerebellum, hematoma, intracerebellar
3	1.6%	0	0.0%	0	0.0%	3	1.1%	cerebellum, hematoma, subdural
0	0.0%	4	5.2%	0	0.0%	4	1.5%	cerebrum, contusion(s)
1	0.5%	0	0.0%	0	0.0%	1	0.4%	cerebrum, diffuse axonal injury
24	13.2%	12	15.6%	3	21.4%	39	14.3%	cerebrum, hematoma, epidural or extradural
22	12.1%	8	10.4%	1	7.1%	31	11.4%	cerebrum, hematoma, intracerebral
30	16.5%	11	14.3%	3	21.4%	44	16.1%	cerebrum, hematoma, subdural
13	7.1%	8	10.4%	2	14.3%	23	8.4%	cerebrum, edema
4	2.2%	3	3.9%	1	7.1%	8	2.9%	cerebrum, intraventricular hemorrhage
11	6.0%	3	3.9%	1	7.1%	15	5.5%	base or vault fracture
2	1.1%	1	1.3%	1	7.1%	4	1.5%	unconscious on admission or initial observation at scene, no other injury description
**122**	**67.0%**	**54**	**70.1%**	**13**	**92.9%**	**189**	**69.2%**	**head, total**

								**face**
1	0.5%	2	2.6%	0	0.0%	3	1.1%	maxilla fracture, blood loss > 20% by volume
**1**	**0.5%**	**2**	**2.6%**	**0**	**0.0%**	**3**	**1.1%**	**face, total**

								**neck**
1	0.5%	0	0.0%	0	0.0%	1	0.4%	carotid, laceration, major
**1**	**0.5%**	**0**	**0.0%**	**0**	**0.0%**	**1**	**0.4%**	**neck total**

								**thorax**
2	1.1%	0	0.0%	0	0.0%	2	0.7%	(crush) bilateral obliteration of a substantial portion of the chest cavity including internal organs
1	0.5%	0	0.0%	0	0.0%	1	0.4%	aorta, thoracic, laceration, major, with hemorrhage
0	0.0%	1	1.3%	0	0.0%	1	0.4%	heart (myocardium), contusion, severe
11	6.0%	3	3.9%	0	0.0%	14	5.1%	lung, contusion, bilateral
0	0.0%	2	2.6%	0	0.0%	2	0.7%	lung, laceration, bilateral with hemomediastinum
18	9.9%	9	11.7%	0	0.0%	27	9.9%	rib cage, fracture > 3 ribs on one side at least, with hemo-/pneumothorax
7	3.8%	3	3.9%	0	0.0%	10	3.7%	rib cage, fracture, with flail
**39**	**21.4%**	**18**	**23.4%**	**0**	**0.0%**	**57**	**20.9%**	**thorax, total**

								**abdomen**
1	0.5%	0	0.0%	0	0.0%	1	0.4%	anus, laceration, massive
1	0.5%	1	1.3%	0	0.0%	2	0.7%	kiney, laceration, major
1	0.5%	0	0.0%	0	0.0%	1	0.4%	liver laceration, massive, complex
7	3.8%	0	0.0%	0	0.0%	7	2.6%	spleen laceration
0	0.0%	0	0.0%	1	7.1%	1	0.4%	stomach laceration, massive
**10**	**5.5%**	**1**	**1.3%**	**1**	**7.1%**	**12**	**4.4%**	**abdomen, total**

								**cervical spine**
2	1.1%	1	1.3%	0	0.0%	3	1.1%	brachial plexus injury
4	2.2%	0	0.0%	0	0.0%	4	1.5%	cord contusion, laceration or complete cord syndrom
**6**	**3.3%**	**1**	**1.3%**	**0**	**0.0%**	**7**	**2.6%**	**cervical spine, total**

								**thoracic spine**
2	1.1%	1	1.3%	0	0.0%	3	1.1%	complete cord syndrom, with fracture
**2**	**1.1%**	**1**	**1.3%**	**0**	**0.0%**	**3**	**1.1%**	**thoracic spine, total**

								**lower extremities**
1	0.5%	0	0.0%	0	0.0%	1	0.4%	pelvis, substantial deformation and displacement with associated vascular disruption or with major retroperitoneal hematoma
**2**	**1.1%**	**0**	**0.0%**	**0**	**0.0%**	**2**	**0.7%**	**lower extremities, total**

The injury patterns observed here are fairly similar to those observed in New Zealand [21], if for head injuries we count the internal organ injuries together with the unspecified head injuries (unconsciousness, without any further injury description), and take account of the fact that we have only displayed AIS 2+ injuries. We have excluded AIS 1 injuries, firstly because they are really minor, being predominantly contusions and abrasions, and secondly, because there are so many of them that they would obscure the more serious injuries.

### Strengths and weaknesses

This study has a number of strengths. First of all, it is based on a very large number of subjects, which confers high statistical power. Second, and more importantly, these subjects were identified using a road trauma registry which covers outpatients, inpatients and fatalities. In other words, the study covers the whole range of injury severities. There might be some selection bias among the slightly injured (MAIS 1) casualties; nevertheless, the coverage rate of the registry is estimated at 80% [5]: the study sample is close to completeness and therefore to representativity.

Another way of assessing the representativity of this study is to compare it with other studies. The proportion of injured cyclists who were involved in a collision with a motor vehicle observed here was similar to that observed in other countries in studies that are based on medical data. For instance, in a Canadian study and an American study, the proportions were 31% and 30% respectively among hospitalized cyclists [17,22]. In an Australian study, 8% of cycling children treated in emergency departments [23] had collided with a motor vehicle.

On the contrary, the results of our study are very different from one in Germany in which two-thirds of the injured cyclists had collided with a motor vehicle [24]. However this study was based on police crash data. French police data describes a similar situation: in 2006, 88.4% of cyclist crashes in the police reports involved another vehicle [25]. This is due to the very low reporting of minor and/or of bicycle-only crashes in police data [5]. In short, our study matches other studies that are based on medical data well.

Each injury was coded with the Abbreviated Injury Scale, and there was no limit to the number of injuries per casualty. The Abbreviated Injury Scale provides a precise description of injured body regions, injury types and injury severity.

Another strength of this study is that it takes account of different cyclist types. The idea was to distinguish between cyclists according to their type of bicycle use, namely for sports or leisure purposes or as a means of transport. One weakness is that we only have an approximation of this using age and crash location (in towns vs outside towns). The approximation seems, however, to be valid since the three groups exhibit different crash patterns and these are consistent with the cycling patterns of sports and non-sport cyclists as measured by the French national travel survey [12].

Moreover, our classification of cyclists according to their crash location ("outside towns" vs "in towns"), means that our results are not affected by the urbanization rate of the Rhône county (which is rather high), and can hence be somewhat generalised.

A weakness of the study (common to many studies on cycling injuries) is the lack of exposure data, i.e. data on bicycle use. Distributions of injured cyclists according to crash circumstances (month, weekday/week-end, night/day...) mostly reflect the pattern of cycling use. If we had data on cycling use, we could estimate the risk of crash and therefore identify the situations or groups of cyclists that are most at risk. For instance, is the probability of a crash higher in urban settings than in rural settings? one could imagine so because of more traffic and hence higher probability of conflicts; if it is higher, by how much? Does the crash risk depend on the type of road? We need such knowledge before setting bicycle safety policies.

The next step will be to obtain exposure data. This should be feasible through the existence of regional mobility surveys. The surveys on the areas of Lille and Grenoble have already been used to estimate the amount of travelled kilometres according to road user type [26,27]. Crash risks were further estimated, but crash data came from the police. It is planned to use the mobility survey of Lyon together with the crash data from the Rhône registry to estimate crash risks in cyclists.

The distributions of injured cyclists are nevertheless informative, in particular for exploring the differences between the cyclists' types.

## Conclusion

This descriptive study yields some interesting results. First of all, cycling type appears relevant for studying bicycle crashes and injuries. Secondly, collisions with a motor-vehicle were not the most frequent crashes. The proportion of such crashes varies with cycling type: it is 8% for injured children, 17% for teenagers and adults injured outside towns and up to 31% for those injured in towns.

The severity of injuries and injury patterns are quite different between bicycle-only crashes and collisions with a motor vehicle: the latter are associated with more injuries to the lower extremities and, more importantly, injuries that are more serious, with a higher percentage of internal organ injuries. These probably match to a first blow on the legs of the cyclists (by the motor-vehicle), followed by a fall where the head is most often injured.

As a whole, children are the less severely injured, followed by 'teenagers and adults injured in towns' with the highest severity for 'teenagers and adults injured outside towns'. This applies both to collisions with a motor vehicle and bicycle-only crashes. This is probably due to lower speeds in towns, among both motor vehicles and cyclists themselves (as opposed to rural areas, where the sports cyclists reach relatively high speeds, as well as motor vehicles of course).

This indicates that lowering the speeds could be a target of bicycle safety policies. Also, bicycle safety policies should account for the different types of cyclists. However, additional recommendations about bicycle safety policies can not be given before exposure data can be obtained and used in the estimation of crash risks.

## List of abbreviations used

AIS: Abbreviated Injury Scale; MAIS: Maximum AIS = AIS severity score of the most severe injury; ZAUER: Zones d'Aire Urbaine et de l'Espace Rural = Zoning of Urban Areas and Rural Space

## Competing interests

The authors declare that they have no competing interests.

## Authors' contributions

EA participated in the design of the study, conducted the statistical analysis, and drafted the manuscript. MC participated in the design of the study, in the interpretation of results and helped to draft the manuscript. BT and BL initiated the study, participated in the design and in the supervision. All the authors read and approved the final manuscript.

## Pre-publication history

The pre-publication history for this paper can be accessed here:

http://www.biomedcentral.com/1471-2458/11/653/prepub
